# Supplementary material for: Endophytic Bacteria from the Desiccation-Tolerant Plant Selaginella lepidophylla and Their Potential as Plant Growth-Promoting Microorganisms
Source: Microorganisms. 2024 Dec 21;12(12):2654. doi: 10.3390/microorganisms12122654 (PMC11728030; doi:10.3390/microorganisms12122654)
Supplement: Supplementary file 1 [file microorganisms-12-02654-s001.zip › microorganisms-3349137-supplementary.pdf]

Supplementary Table S1. Plant growth promotion parameters of bacterial isolates of *Selaginella lepidophylla*.

Name	ERIC-PCR	Molecular identification 16S rRNA / blast access number	Accession number of our bacterial isolate	IAA (µg/ml) With Trp 24h	IAA (µg/ml) With Trp 48h	PS mm	BNF +/-	Trehalose µg/ml	Siderophores mm – colors (pink, P; orange, O)
Azospirillum brasilense Cd	Control	ND	ND	9.35	26.98	-	+	2.58	0
Pseudomonas lutea	Control	ND	ND	2.09	0.7	ND	ND	ND	ND
Arthrobacter chlorophenolicus 30.16	Control	ND	ND	ND	ND	ND	ND	48.77	ND
**Bacterial isolated of *Selaginella lepidophylla* in the hydrated state (rainy season).**									
Nutrient Agar Medium (AN) microphyll									
1. SlL1	Pattern 12	*Pantoea* sp. / CP028349.1	PQ497908	46.38	50.58	2.67	+	11.82	2.08 – O
2. SlL2	Pattern 1	*Pseudomonas* sp. / MT367856.1	PQ497909	7.89	9.59	2.46	+	4.8	2.17 - O
3. SlL3	Pattern 9	ND	ND	0	9.04	-	-	4.03	ND
4. SlL4	Pattern 10	*Pantoea* sp. / MN006192.1	PQ497910	46.42	51.33	2.87	+	21.25	2.29 - P
5. SlL5	Pattern 4	*Pseudomonas* sp. / MZ339754.1	PQ497911	10.16	19.29	2.5	+	12.19	2.11 – O
6. SlL6	Pattern 105	*Pseudomonas* sp. / MH725608.1	PQ497912	2.77	13.15	2.31	-	1.88	0
7. SlL7	Pattern 106	ND	ND	0	0.58	2.37	+	7.56	ND
8. SlL8	Pattern 107	ND	ND	0	16.02	X	NC	3.38	ND
Nutrient Agar Medium (AN) rhizophore									
9. SlL9	Pattern 13	*Rhizobium* sp. / MK574776.1	PQ497913	0	37.17	2.47	-	4.72	0
10. SlL10	Pattern 17	*Pseudomonas* sp. / MK446732.1	PQ497914	3.44	2.49	2.44	+	35.75	2.28 - P
11. SlL11	Pattern 15	*Pseudomonas* sp. / MH341938.1	PQ497915	1.05	4.4	3.29	+	4.86	2.18 – O
12. SlL12	Pattern 11	*Pantoea* sp. / MF807973.1	PQ497916	46.75	49.56	2.54	+	25.01	2.45 – P
13. SlL13	Pattern 8	ND	ND	0	0	2.73	+	7.63	ND
14. SlL14	Pattern 1	*Pseudomonas* sp. / MT981740.1	PQ497917	6.49	7.14	2.55	+	6.42	2.08 – O
15. SlL15	Pattern 4	ND	ND	0	0	2.19	-	1.41	ND
16. SlL16	Pattern 16	ND	ND	2.54	25.85	-	+	0.82	ND
17. SlL17	Pattern 14	ND	ND	0	0	-	-	1.41	ND
18. SlL18	Pattern 18	*Streptomyces* sp. / DQ026651.1	PQ541125	0	38.93	2.32	+	7.32	0
19. SlL19	Pattern 6	ND	ND	0	45.42	-	+	1.43	ND
20. SlL20	Pattern 6	*Streptomyces* sp. / KY352802.1	PQ541126	1.33	44.45	2.26	+	5.95	2.15 - O
21. SlL21	Pattern 5	ND	ND	0	0	2.36	+	7.71	ND
22. SlL22	Pattern 2	*Pseudomonas* sp. / OM985542.1	PQ570561	5.33	0.3	-	+	18.31	ND
23. SlL23	Pattern 2	*Pseudomonas* sp. / MW959056.1	PQ497918	5.87	0.05	-	+	18.73	2.11 – O
24. SlL24	Pattern 7	*Pantoea* sp. / MG557675.1	PQ497919	0	4.53	3.51	+	13.79	2.05 – O
25. SlL25	Pattern 7	*Pseudomonas* sp. / MH734763.1	PQ497920	46.15	48.29	2.49	+	3.96	2.04 – O
26. SlL26	Pattern 108	*Pseudomonas* sp. / MT626824.1	PQ497921	3.08	22.4	2.34	+	6.1	0
27. SlL27	Pattern 107	ND	ND	0	1.66	2.24	-	1.44	ND
28. SlL28	Pattern 106	ND	ND	0	15.29	-	NC	ND	ND
Medium Luria Bertani (LB) microphyll									
29. SlL29	Pattern 3	*Pseudomonas* sp. / KR267325.1	PQ497922	6.83	5.53	2.52	+	15.52	0
30. SlL30	Pattern 28	*Rhizobium* sp. / MH769523.1	PQ497923	1.39	5	2.2	+	5.22	0
31. SlL31	Pattern 26	ND	ND	0	3.28	2.38	+	4.29	ND
32. SlL32	Pattern 24	ND	ND	2.57	0	2.25	-	1.48	ND
33. SlL33	Pattern 31	*Pseudomonas* sp. / CP103374.1	PQ497924	0	32.93	2.65	+	11.32	2.15 – O
34. SlL34	Pattern 30	ND	ND	0	5.89	2.27	+	6.04	ND
35. SlL35	Pattern 29	ND	ND	4.85	0.03	-	-	0.67	ND
36. SlL36	Pattern 23	*Pseudomonas* sp. / MZ339754.1	PQ497925	2.08	1.96	2.19	+	7.86	2.17 – O
37. SlL37	Pattern 3	ND	ND	12.01	9.55	2.63	+	ND	ND
38. SlL38	Pattern 19	*Pseudomonas* sp. / MW019517.1	PQ497926	6.11	7.24	2.35	+	2.84	0
39. SlL39	Pattern 27	*Pseudomonas* sp. / MT089709.1	PQ497927	0.24	8.81	2.26	+	5.95	2.23 – O
40. SlL40	Pattern 25	*Pseudomonas* sp. / MT626824.1	PQ497928	0	0	2.27	+	2.64	0
41. SlL41	Pattern 111	ND	ND	0	1.45	2.37	+	5.48	ND
42. SlL42	Pattern 111	ND	ND	0	3.11	2.19	+	0.21	ND
Medium Luria Bertani (LB) rhizophore									
43. SlL43	Pattern 20	ND	ND	0	50.02	2.17	+	15.62	ND
44. SlL44	Pattern 21	*Pantoea* sp. / JN853256.1	PQ497929	47.87	55.13	2.47	+	24.56	2.08 – O
45. SlL45	Pattern 32	ND	ND	0	13.68	-	+	17.22	ND
46. SlL46	Pattern 22	*Pantoea* sp. / MF807973.1	PQ497930	16.43	16.07	2.17	+	2.34	2.06 – O
47. SlL47	Pattern 52	*Pantoea* sp. / KF447415.1	PQ497931	0.58	10.77	2.26	+	2.76	2.18 – O
48. SlL48	Pattern 109	ND	ND	0	3.49	2.33	+	10.24	ND
49. SlL49	Pattern 107	ND	ND	0	0	-	-	2.67	ND
Medium Peptone Yeast Extract (PY) microphyll									
50. SlL50	Pattern 56	*Pseudomonas* sp. / MT626824.1	PQ497932	0	3.62	-	+	20.01	0
51. SlL51	Pattern 57	*Pseudomonas* sp. / MT626824.1	PQ497933	0	3.04	2.33	+	22.17	0
52. SlL52	Pattern 70	ND	ND	0	0	2.47	+	19.33	ND
53. SlL53	Pattern 61	ND	ND	4.16	0	-	+	0.003	ND
54. SlL54	Pattern 57	ND	ND	0	0	2.22	+	1.46	ND
55. SlL55	Pattern 68	*Pantoea* sp. / MN914042.1	PQ497934	35.82	51.53	2.42	+	25.75	2.06 – O
56. SlL56	Pattern 67	ND	ND	32.04	20.48	-	+	-	ND
57. SlL57	Pattern 69	ND	ND	28.41	19.65	-	+	-	ND
58. SlL58	Pattern 57	ND	ND	22.35	46.25	-	+	7.25	ND
59. SlL59	Pattern 61	*Curtobacterium* sp. / MG778772.1	PQ497935	13.69	52.28	-	+	ND	2.12 – O
60. SlL60	Pattern 61	ND	ND	8.82	49.69	-	+	4.27	ND
61. SlL61	Pattern 43	*Pseudomonas* sp. / MZ339754.1	PQ497936	0.77	10.47	2.25	+	6.52	2.07 – O
62. SlL62	Pattern 74	*Pseudomonas* sp. / MT949893.1	PQ497937	0.82	5.25	2.8	+	1.84	2.17 – O
63. SlL63	Pattern 72	ND	ND	1.39	1	-	+	7.12	ND
64. SlL64	Pattern 61	ND	ND	8.6	1.25	-	+	7.03	ND
65. SlL65	Pattern 43	ND	ND	0	1.22	2.41	+	ND	ND
66. SlL66	Pattern 63	ND	ND	31.21	37.05	2.19	+	ND	ND
67. SlL67	Pattern 63	*Pantoea* sp. / JN853256.1	X falta	40.57	38.84	2.43	+	29.5	2.06 – O
68. SlL68	Pattern 54	ND	ND	21.03	49.42	-	+	-	ND
69. SlL69	Pattern 58	*Curtobacterium* sp. / MT487608.1	PQ497938	1.35	36.55	2.98	-	-	0
70. SlL70	Pattern 59	*Pseudomonas* sp. / ON763830.1	PQ497939	1.41	36.5	2.33	+	1.18	3.95 – O
71. SlL71	Pattern 107	ND	ND	0	0	2.31	+	2.9	ND
72. SlL72	Pattern 107	ND	ND	1.37	4.29	-	+	0.36	ND

73.	SlL73	Pattern 110	ND	ND	0.16	0.35	X	NC	ND	ND
Medium Peptone Yeast Extract (PY) rhizophore										
74.	SlL74	Pattern 71	ND	ND	10.24	1.29	-	+	2.45	ND
75.	SlL75	Pattern 65	ND	ND	2.93	13.73	2.37	+	ND	ND
76.	SlL76	Pattern 75	ND	ND	0	4.09	-	+	ND	ND
77.	SlL77	Pattern 60	ND	ND	0	1.26	-	-	2.01	ND
78.	SlL78	Pattern 76	ND	ND	0	0	-	+	ND	ND
79.	SlL79	Pattern 53	ND	ND	0	0	2.37	+	0.88	ND
80.	SlL80	Pattern 51	ND	ND	0	1.75	-	+	-	ND
81.	SlL81	Pattern 62	ND	ND	0	6.93	-	+	ND	ND
82.	SlL82	Pattern 73	ND	ND	0	2.16	-	-	0.08	ND
83.	SlL83	Pattern 55	ND	ND	0	6.16	-	-	-	ND
84.	SlL84	Pattern 78	ND	ND	0	1.45	-	+	0.99	ND
85.	SlL85	Pattern 66	ND	ND	0	1.95	-	+	-	ND
86.	SlL86	Pattern 49	ND	ND	0.53	0	-	+	ND	ND
87.	SlL87	Pattern 49	ND	ND	0.11	0	-	+	ND	ND
88.	SlL88	Pattern 49	ND	ND	0.28	0.28	-	+	6.12	ND
89.	SlL89	Pattern 64	*Pantoea* sp. / KC759399.1	PQ497940	19.37	14.57	2.18	+	1.9	2.10 – O
90.	SlL90	Pattern 112	ND	ND	0	2.52	-	+	-	ND
91.	SlL91	Pattern 75	*Burkholderia* sp. / MN272283.1	PQ497941	6.58	0	3.67	+	11.45	4.04 – P
92.	SlL92	Pattern 62	ND	ND	0	0	2.3	+	4.31	ND
93.	SlL93	Pattern 107	ND	ND	0.33	4.09	-	+	ND	ND
94.	SlL94	Pattern 110	ND	ND	0	0.99	-	-	ND	ND
95.	SlL95	Pattern 110	ND	ND	0	0	-	-	ND	ND
Potato Dextrose Agar Medium (PDA) microphyll										
96.	SlL96	Pattern 33	*Pseudomonas* sp. / KR267326.1	PQ497942	1.25	3.8	-	-	31.03	0
97.	SlL97	Pattern 34	*Pseudomonas* sp. / MT218359.1	PQ497943	0	3.58	-	+	24.21	2.12 – O
98.	SlL98	Pattern 40	ND	ND	0	6.5	2.9	+	ND	ND
100.	SlL100	Pattern 35	ND	ND	0.91	11.16	-	+	3.25	ND
101.	SlL101	Pattern 40	ND	ND	3.05	7.83	2.67	+	ND	ND
102.	SlL102	Pattern 42	ND	ND	0.17	5.02	3.08	+	-	ND
103.	SlL103	Pattern 35	ND	ND	10.39	0	-	+	ND	ND
104.	SlL104	Pattern 77	*Pseudomonas* sp. / MZ339754.1	PQ497944	1.28	9.74	2.58	+	0.4	2.23 – O
105.	SlL105	Pattern 44	ND	ND	0	3.73	2.27	+	ND	ND
106.	SlL106	Pattern 41	*Pseudomonas* sp. / MT981740.1	PQ497945	5.59	0	-	+	ND	2.22 – O
107.	SlL107	Pattern 44	*Pseudomonas* sp. / MZ339754.1	PQ497946	2.48	3.12	2.35	+	10.11	2.16 – O
108.	SlL108	Pattern 36	ND	ND	0.68	1.59	2.96	-	-	ND
109.	SlL109	Pattern 113	*Pseudomonas* sp. / MZ339754.1	PQ497947	3.28	12.93	2.46	+	6.6	3.91 – O
110.	SlL110	Pattern 65	*Pseudomonas* sp. / KM434836.1	PQ497948	8.92	18.43	2.22	+	1.1	0
111.	SlL111	Pattern 110	*Curtobacterium* sp. / MT487608.1	PQ497949	0	0.7	2.23	+	0.03	0
112.	SlL112	Pattern 110	ND	ND	0	2.18	-	NC	ND	ND
Potato Dextrose Agar Medium (PDA) rhizophore										
113.	SlL113	Pattern 45	*Pseudomonas* sp. / MT089709.1	PQ497950	1.77	3.77	2.35	+	5.51	4.25 – O
114.	SlL114	Pattern 38	*Pseudomonas* sp. / KU519713.1	PQ497951	29.1	34.34	3.42	+	12.88	0
115.	SlL115	Pattern 46	ND	ND	17.07	24.49	2.32	+	ND	ND
116.	SlL116	Pattern 47	*Pseudomonas* sp. / KC822776.1	PQ497952	3.05	6.51	2.67	+	-	4.60 – O
117.	SlL117	Pattern 37	ND	ND	0	2.56	-	+	10.18	ND
118.	SlL118	Pattern 114	ND	ND	0.97	3.07	-	NC	ND	ND
119.	SlL119	Pattern 48	ND	ND	0.46	4.22	-	+	15.78	ND
120.	SlL120	Pattern 49	ND	ND	0	0.32	-	+	ND	ND
121.	SlL121	Pattern 50	ND	ND	3.07	0	3.83	+	13.51	ND
122.	SlL122	Pattern 39	ND	ND	17.03	36.34	-	+	3.87	ND
123.	SlL123	Pattern 49	ND	ND	0	2.08	-	+	ND	ND
**Bacterial isolated of *Selaginella lepidophylla* in the dehydrated state (drought season).**										
Nutrient Agar Medium (AN) microphyll										
1.	SlS1	Pattern 88	*Erwinia* sp. / MN733145.1	PQ497953	41.77	49.16	3.39	+	13.14	2.08 – O
2.	SlS2	Pattern 84	*Agrobacterium* sp. / MT422168.1	PQ570562	24.39	43.63	-	+	0.02	ND
3.	SlS3	Pattern 90	*Pantoea* sp. / MN758864.1	PQ497954	39.29	46.78	2.74	+	18.11	2.04 – O
4.	SlS4	Pattern 100	*Pseudomonas* sp. / MW959056.1	PQ497955	2.69	3.83	-	+	19.3	2.09 – O
5.	SlS5	Pattern 98	*Pseudomonas* sp. / MZ339754.1	PQ497956	1.05	11.7	2.34	+	6.37	2.12 – O
Nutrient Agar Medium (AN) rhizophore										
6.	SlS6	Pattern 99	ND	ND	0	0	-	+	ND	ND
7.	SlS7	Pattern 102	ND	ND	0	0	-	-	ND	ND
8.	SlS8	Pattern 89	ND	ND	0	0	-	+	ND	ND
Medium Luria Bertani (LB) microphyll										
9.	SlS9	Pattern 101	*Enterobacter* sp. / MT505114.1	PQ497957	44.44	50.73	3.18	+	9.3	2.04 – O
10.	SlS10	Pattern 87	*Agrobacterium* sp. / MT409553.1	PQ497958	25.68	40.38	-	+	ND	0
11.	SlS11	Pattern 79	ND	ND	14.25	18.73	2.41	+	-	ND
12.	SlS12	Pattern 86	ND	ND	24.41	17.61	-	+	0.07	ND
13.	SlS13	Pattern 79	ND	ND	18.82	34.92	-	+	0.59	ND
14.	SlS14	Pattern 101	*Pseudomonas* sp. / MZ339754.1	PQ497959	1.24	30.02	2.26	+	ND	2.15 – O
15.	SlS15	Pattern 101	*Pantoea* sp. / MN758864.1	PQ497960	30.31	38.47	2.42	+	ND	2.02 – O
Medium Luria Bertani (LB) rhizophore										
16.	SlS16	Pattern 83	ND	ND	10.88	26.49	-	-	-	ND
17.	SlS17	Pattern 74	ND	ND	-	-	X	NC	ND	ND
18.	SlS18	Pattern 15	ND	ND	9.3	18.33	-	+	ND	ND
Medium Peptone Yeast Extract (PY) microphyll										
19.	SlS19	Pattern 74	ND	ND	19.14	11.52	-	+	0.04	ND
20.	SlS20	Pattern 79	ND	ND	10.34	32.91	-	+	ND	ND
21.	SlS21	Pattern 94	*Pseudomonas* sp. / MT626824.1	PQ497961	0	0.71	-	+	5.53	0
22.	SlS22	Pattern 92	ND	ND	0	0	-	+	ND	ND
23.	SlS23	Pattern 93	ND	ND	0	11.04	-	+	0.42	ND
24.	SlS24	Pattern 96	ND	ND	0	0	-	+	ND	ND
25.	SlS25	Pattern 74	ND	ND	11.92	7.14	-	+	ND	ND
26.	SlS26	Pattern 96	*Pantoea* sp. / MN758864.1	PQ497962	34.69	47.88	2.96	+	15.66	2.08 – O

27.	SlS27	Pattern 87	*Pantoea* sp. / OK325604.1	PQ497963	41.66	47.58	2.33	+	13.97	2.02
			Medium Peptone Yeast Extract (PY) rhizophore							
28.	SlS28	Pattern 81	*Rhizobium* sp. / MT534119.1	PQ497964	8.27	41	-	+	1.35	2.05 – O
29.	SlS29	Pattern 104	ND	ND	25.18	45.87	-	-	0.21	ND
30.	SlS30	Pattern 80	ND	ND	24.96	41.7	-	+	0.32	ND
31.	SlS31	Pattern 82	ND	ND	0	42.16	-	+	-	ND
32.	SlS32	Pattern 85	ND	ND	20.89	43.52	-	+	0.02	ND
33.	SlS33	Pattern 87	ND	ND	-	-	-	NC	ND	ND
34.	SlS34	Pattern 87	ND	ND	-	-	-	NC	ND	ND
35.	SlS35	Pattern 87	ND	ND	0	6.38	-	+	0.03	ND
			Potato Dextrose Agar Medium (PDA) microphyll							
36.	SlS36	Pattern 90	*Pseudomonas* sp. / MZ339754.1	PQ497965	5.66	17.22	2.37	+	ND	2.08 – O
37.	SlS37	Pattern 74	ND	ND	-	-	2.32	NC	ND	ND
38.	SlS38	Pattern 97	*Pseudomonas* sp. / MZ339754.1	PQ497966	0	23.03	2.36	+	5.32	0
39.	SlS39	Pattern 91	*Pseudomonas* sp. / CP052860.1	PQ497967	33.61	41.85	2.29	+	ND	2.40 – P
			Potato Dextrose Agar Medium (PDA) rhizophore							
40.	SlS40	Pattern 95	ND	ND	-	-	2.97	+	ND	ND
41.	SlS41	Pattern 103	ND	ND	-	-	-	NC	ND	ND

Supplementary Table S1. Plant growth promotion parameters of bacterial isolates of *Selaginella lepidophylla*. Shows the name of the isolate, analysis of enterobacterial repetitive intergenic consensus sequences (ERIC-PCR), molecular identification by 16S rRNA and blast number accession, accession number of our bacterial isolate, production of indole acetic acid (IAA) in JP medium with Trp at 24 and 48 h, phosphate solubilization (PS), biological nitrogen fixation (BNF), trehalose quantification, and siderophore production. Positive result (+); negative result (-); Did not grow (NC); Not determined (ND); Not present (NP). According to the statistical analyses, significant differences were presented for the IAA production experiments at 24 and 48 hours in the JP culture medium with Trp for the rainy season: $F = 134.24$ and $F = 101.25$ for the low IAA producers. $F=216.58$ and $F= 68.21$ for medium AIA producers and $F= 928.53$ and $F= 743.69$ for high AIA producers. And for the drought season: $F=846.92$ and $F=83.27$ for low IAA producers, $F=262.4$ and $F=235.02$ for medium IAA producers and $F= 586.38$ and $F= 703$ for high IAA producers. In the PS: $F=14.43$ and $F= 6.69$ for the rainy and drought seasons, respectively. And for the production of siderophores: $F=117.66$ and $F=3959.29$ for the rainy and drought seasons, respectively. $Pr>F= <0.0001$ and $\alpha=0.05$.
